# Characterizing the Physical Properties and Cell Compatibility of Phytoglycogen Extracted from Different Sweet Corn Varieties

**DOI:** 10.3390/molecules25030637

**Published:** 2020-02-01

**Authors:** Renjie Liu, Susan K. Boehlein, William F. Tracy, Marcio F. R. Resende, Gregory A. Hudalla

**Affiliations:** 1 J. Crayton Pruitt Family Department of Biomedical Engineering, Wertheim College of Engineering, University of Florida, Gainesville, FL 32611, USA; renjieliu@ufl.edu; 2 Horticultural Sciences Department, University of Florida, Gainesville, FL 32611, USA; sboehlei@ufl.edu; 3 Department of Agronomy, University of Wisconsin-Madison, Madison, WI 53706, USA; wftracy@wisc.edu

**Keywords:** polysaccharide, phytoglycogen, cytocompatibility

## Abstract

Owing to its unique structure and properties, the glucose dendrimer phytoglycogen is gaining interest for medical and biotechnology applications. Although many maize variants are available from commercial and academic breeding programs, most applications rely on phytoglycogen extracted from the common maize variant, *sugary1*. Here we characterized the solubility, hydrodynamic diameter, water-binding properties, protein contaminant concentration, and cytotoxicity of phytoglycogens from different maize sources, A632*su1*, A619*su1*, Wesu7, and Ia453*su1*, harboring various *sugary1* mutants. A619*su1*-SW phytoglycogen was cytotoxic while A632*su1*-SW phytoglycogen was not. A632*su1*-Pu phytoglycogen promoted cell growth, whereas extracts from A632*su1*-NE, A632*su1*-NC, and A632*su1*-CM were cytotoxic. Phytoglycogen extracted from Wesu7*su1*-NE using ethanol precipitation was cytotoxic. Acid-treatment improved Wesu7 phytoglycogen cytocompatibility. Protease-treated Wesu7 extracts promoted cell growth. Phytoglycogen extracted from Ia453*su1* 21 days after pollination (“Ia435*su1* 21DAP”) was cytotoxic, whereas phytoglycogen extracted at 40 days (“Ia435*su1* 40DAP”) was not. In general, size and solubility had no correlation with cytocompatibility, whereas protein contaminant concentration and water-binding properties did. A632*su1*-CM had the highest protein contamination among A632 mutants, consistent with its higher cytotoxicity. Likewise, Ia435*su1* 21DAP phytoglycogen had higher protein contamination than Ia435*su1* 40DAP. Conversely, protease-treated Wesu7 extracts had lower protein contamination than the other Wesu7 extracts. A632*su1*-NE, A632*su1*-NC, and A632*su1*-CM had similar water-binding properties which differed from those of A632*su1*-Pu and A632*su1*-SW. Likewise, water binding differed between Ia435*su1* 21DAP and Ia435*su1* 40DAP. Collectively, these data demonstrate that maize phytoglycogen extracts are not uniformly cytocompatible. Rather, maize variant, plant genotype, protein contaminants, and water-binding properties are determinants of phytoglycogen cytotoxicity.

## 1. Introduction

Polysaccharides, which are polymers of carbohydrates, are abundant throughout the natural world. They are found in algae, plants, microbes, bacteria, and animals [1,2]. Depending on their origin, polysaccharides can have unique chemical compositions [3,4], structures [5], and biological functions [6,7], such as cell adhesion [8,9,10], signaling [11,12], and activation [6,13,14,15,16,17]. Understanding the role of polysaccharides within living systems, and establishing methods to extract them, have enabled their increasing use as biomaterials [18,19]. For example, polysaccharides are employed as carriers for sustained drug delivery [20,21], scaffolds for tissue regeneration [22,23], and as protective agents for vaccines and pharmaceuticals [24,25,26]. Central to the success of polysaccharides in these applications is their biocompatibility and biodegradability [27]. 

**Figure 1:**
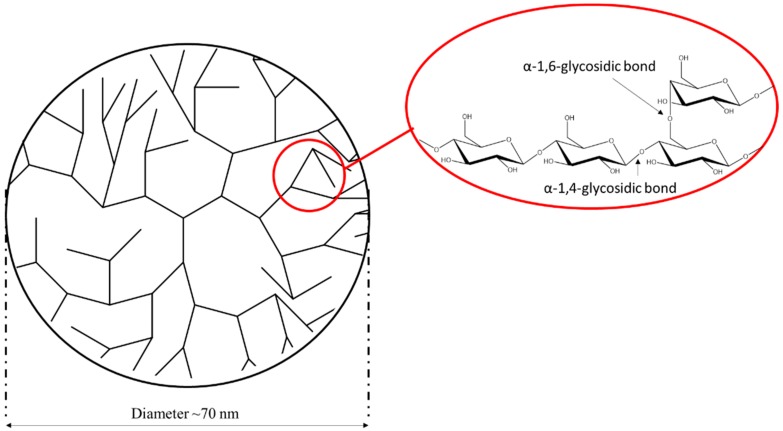
Schematic representation of the structure of phytoglycogen.

Phytoglycogen is a glucose polysaccharide that is produced by plants [28]. Unlike starch, phytoglycogen is a dendritic macromolecule (Figure 1). The extensive branching of phytoglycogen results in unusually high solubility and low viscosity in water [28,29]. These features have led to an increasing interest in phytoglycogen for biomedical and biotechnology applications. For example, it can be used as a food additive [30,31], a vaccine adjuvant [32], an anti-oxidative reagent [33] and even as a natural moisturizer [34]. The most common source for phytoglycogen is the kernel of the maize mutant *sugary1 (su1)*, a primary allele explored in commercial sweet corn production [29,35]. Phytoglycogen extracted from *su1* mutants consists of glucose monomers connected by α-1,4-glycosidic linkages, with branching on about every 13 monomer via an α-1,6-glycosidic linkage [28,36]. Phytoglycogen dispersed in aqueous solutions shows a uniform spherical nanoparticle shape [37,38], and forms an opalescent colloidal dispersion [39]. The accumulation of phytoglycogen correlates with the deficiency of several debranching enzymes [40,41,42]. Plants contain two distinct types of starch debranching enzymes, the isoamylase type, and the pullulanase type. Isoamylases hydrolyze α-1,6 linkages from amylopectin and glycogen, while pullulanase hydrolyzes the same bond in pullulan [43]. In maize, there are three isoamylases (ISA1, ISA2 and ISA3) and one pullulanase (PUL1). In maize, ISA1 is encoded by the *sugary1* (*su1*) gene. There are currently five distinct naturally occurring mutations reported at the *su1* locus. Three of these mutations are caused by single nucleotide polymorphisms (*su1*-NE, *su1*-NC, *su1*-SW), one has a 1.3 kb insertion (*su1*-CM), and one has an unknown genetic mutation that could not be identified within the coding sequence of the gene (*su1*-Pu) [44]. Immunoblots using the α-isoamylase-type (ISA1) antibody indicate that *su1-*CM is a complete loss of function with no protein being produced. The other alleles form a protein, but enzyme activity cannot be detected in potato starch zymograms [45].

Although phytoglycogen cytotoxicity has been characterized before [32], these efforts have primarily involved the polysaccharide extracted from a sweet corn variant with the *su1*-NE allele, which is also known simply as *su1* or *su1-ref* [28,38,46]. In the current study, we extracted and purified phytoglycogens from plants with various natural mutations of *sugary1* in different sweet corn backgrounds using various methods. The cytotoxicity of these variants was characterized using an NIH3T3 fibroblast in vitro model. The influence of the source on the hydrodynamic diameter of phytoglycogen extracts was assessed using Dynamic Light Scattering (DLS). The interaction of water with phytoglycogen extracts from different sources was estimated using Fourier-transform infrared spectroscopy (FTIR). Lastly, the concentration of contaminating proteins in each phytoglycogen extract was measured using the bicinchoninic acid (BCA) assay. Collectively, these experiments identified relationships between cytotoxicity and the maize variant, the plant genotype, and the extraction methods, which will aid in future efforts to select phytoglycogen extracts that are suitable for use as biomaterials. 

## 2. Results and Discussion

### 2.1. Solubility of Phytoglycogen Extracts from Different Sources

Phytoglycogen was extracted from the sweet corn varieties A632, A619, Wesu7, and Ia453 using either ethanol precipitation, ethanol precipitation with deproteinization, or ethanol precipitation with protease treatment. All phytoglycogen extracts were obtained as white powders and were suspended in 1× phosphate-buffered saline (PBS) at a concentration of 20 mg/mL. We noted that all the solutions were milky, and when passed through a 0.22 µm filter, the suspensions remained milky but with an increase in transparency. The concentration of phytoglycogen extract in each filtered suspension was determined by measuring the mass of powder obtained after lyophilization and subtracting the mass of lyophilized PBS vehicle (Table 1). The solubility of phytoglycogen variants ranged from ~15 to 20 mg/mL; however, no significant differences in solubility were observed between extracts with regard to plant variety, *su1* allele, extraction method, or kernel maturity.

### 2.2. Hydrodynamic Diameter of Phytoglycogen Extracts from Different Sources

All phytoglycogen variants had intensity-weighted mean hydrodynamic diameter (i.e., “*Z*-averages”) ranging from ~66–78 nm with low polydispersity indices (Table 2). In general, these data demonstrated that phytoglycogen hydrodynamic diameter does not depend on plant variety, *su1* allele, or harvest time.

### 2.3. Cytotoxicity of Phytoglycogen Extracts from Different Sources

**Figure 2:**
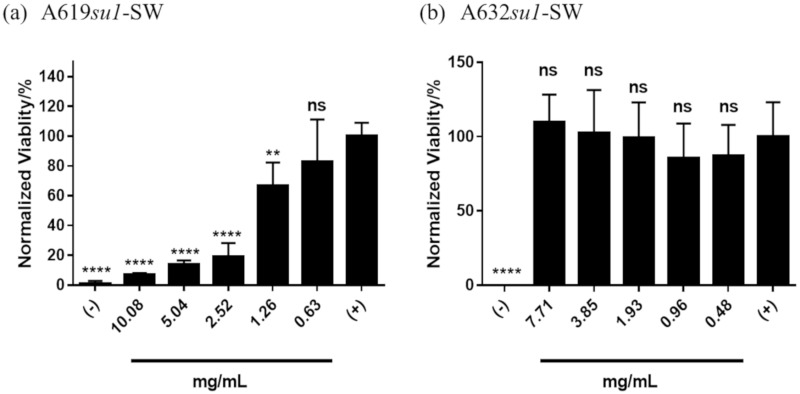
Cytotoxicity of phytoglycogen extracts from two different plant varieties with the *su1*-SW mutant. Phytoglycogen was extracted from both plant varieties using ethanol precipitation. Cells were treated with 50 *v*/*v* % PBS (positive control) or 50 *v*/*v* % DMSO (negative control). Data are presented as mean ± standard deviation (*n* = 5). * indicates statistically significant differences compared to the positive control group; “ns” indicates no difference relative to the positive control group at t = 24 h. Statistical significance was calculated using a one-way ANOVA followed by Tukey’s post-hoc. ** *p* < 0.01 and **** *p* < 0.0001.

**Figure 3:**
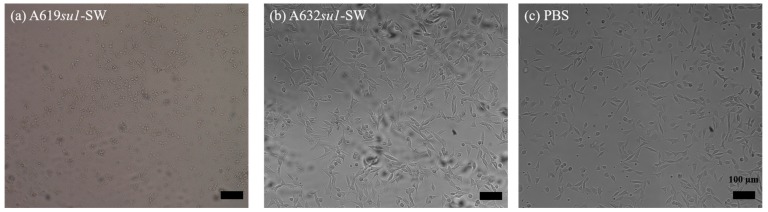
Bright-field micrographs of NIH3T3 cells treated with (**a**) A619*su1*-SW, (**b**) A632*su1*-SW, or (**c**) PBS. Scale bar represents 100 µm in all images.

We characterized the cytotoxicity of phytoglycogen extracts from different sweet corn varieties using different extraction-purification methods by treating NIH3T3 fibroblasts with a range of dilutions of stock extract solutions. First, we compared cells treated with phytoglycogen extracted from 2 different corn lines with the same *su1* allele, *su1*-SW. Both A619*su1-*SW and A632*su1-*SW were extracted using the ethanol precipitation protocol. A619*su1-*SW showed a clear dose-dependent toxicity (Figure 2a), while A632*su1-*SW showed low toxicity at all concentrations tested (Figure 2b). Likewise, cells exposed to A619*su1-*SW had a rounded morphology consistent with dead or dying NIH3T3 fibroblasts, whereas cells exposed to A632*su1-*SW had an elongated morphology consistent with healthy cells and PBS controls (Figure 3). Thus, plants harboring the same *su1* mutant can yield phytoglycogen extracts with different cytotoxicity profiles.

**Figure 4:**
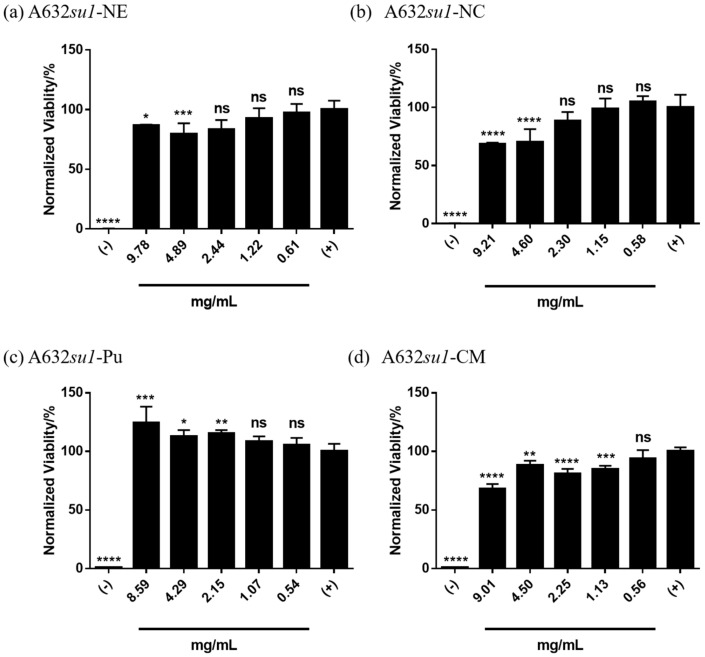
Cytotoxicity of extracts from the same plant with different *su1* mutants. Phytoglycogen was extracted from all plants using ethanol precipitation. Cells were treated with 50 *v*/*v* % PBS (positive control) or 50 *v*/*v* % DMSO (negative control). Data are presented as mean ± standard deviation (*n* = 5). * indicates statistically significant differences compared to the positive control group; “ns” indicates no difference relative to the positive control group at t = 24 h. Statistical significance was calculated using a one-way ANOVA followed by Tukey’s post-hoc. * *p* < 0.05, ** *p* < 0.01, *** *p* < 0.001 and **** *p* < 0.0001.

Next, we compared the cytotoxicity of phytoglycogen extracts from different *su1* mutants in the A632 background. The phytoglycogen extract from A632*su1*-NE was weakly cytotoxic at concentrations ≥ 4.9 mg/mL (Figure 4a), while that from A632*su1*-NC was moderately cytotoxic over the same range (Figure 4b). The phytoglycogen extract from A632*su1*-Pu promoted cell metabolic activity at concentrations ≥ 2.15 mg/mL (Figure 4c), suggesting that fibroblasts can use this variant as an energy source under the tested conditions [47,48]. The phytoglycogen extract from A632*su1*-CM was moderately cytotoxic at concentrations ≥ 1.1 mg/mL (Figure 4d). There was no clear relationship between the type of mutation and the cytotoxicity of phytoglycogen produced. In particular, among the single nucleotide polymorphisms, *su1*-SW was non-toxic, *su1*-NE was weakly toxic, and *su1*-NC was moderately toxic. *su1*-CM, which is characterized by a large insertion and complete loss of ISA1 production, was also moderately toxic. Given that *su1*-NC results in production of ISA1, albeit a non-functional variant, these observations suggested that cytotoxicity is not correlated with the presence or absence of ISA1 in the plant. Taken together with observations from the A632*su1*-SW group (Figure 2b), these results demonstrate that plants harboring different *su1* mutations can yield phytoglycogen extracts with widely varying cytocompatibility, although cytotoxicity cannot be predicted from the type of mutation alone. 

**Figure 5:**
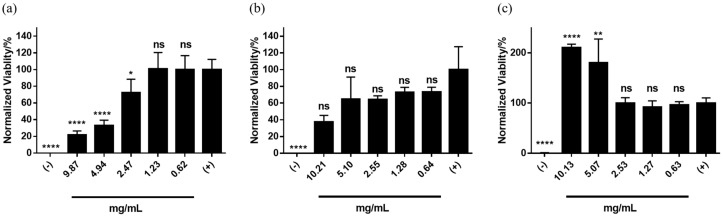
Toxicity of phytoglycogen extracts isolated from Wesu7 using different methods. (**a**) ethanol precipitation; (**b**) ethanol precipitation with deproteinization, and (**c**) ethanol precipitation with protease treatment. Cells were treated with 50 *v*/*v* % PBS (positive control) or 50 *v*/*v* % DMSO (negative control). Data are presented as mean ± standard deviation (*n* = 5). * indicates statistically significant differences compared to the positive control group; “ns” indicates no difference relative to the positive control group at t = 24 h. Statistical significance was calculated using a one-way ANOVA followed by Tukey’s post-hoc. * *p* < 0.05, ** *p* < 0.01, and **** *p* < 0.0001.

Next, we evaluated the cytotoxicity of phytoglycogen extracts isolated from the Wesu7 variety (*su1-*NE allele) using different methods. The different extraction methods did not alter the hydrodynamic diameter of phytoglycogen extracts (Table 2 and Table S1). Phytoglycogen extracts isolated using ethanol precipitation induced significant cell death at concentrations ≥ 2.47 mg/mL (Figure 5a), whereas extracts isolated using ethanol precipitation with acid deproteination induced less cell death over the entire concentration range (Figure 5b). In contrast, phytoglycogen extracts isolated using ethanol precipitation and protease treatment strongly stimulated cell metabolic activity at concentrations ≥ 5 mg/mL (Figure 5c). Collectively, these data further supported the observation that the maize line is an important determinant of phytoglycogen cytotoxicity, given that extracts from the Wesu7 variety with the *su1*-NE allele that were isolated with ethanol precipitation were more cytotoxic than extracts from the A632 variety with the same allele that were isolated with the same method. These data also suggested that extraction method can alter cytotoxicity, as demonstrated by the weakened cytotoxicity of Wesu7 extracts that were deproteinated after ethanol precipitation. This suggests that a similar extraction protocol could potentially decrease the cytotoxicity of phytoglycogen extracts from A619*su1*-SW, A632*su1*-NE, A632*su1*-NC, or A632*su1*-CM, which we will explore with future crop harvests. Unexpectedly, protease treatment yielded phytoglycogen extracts from Wesu7 that stimulated cell growth more potently than extracts from A632*su1*-Pu (Figure 5c and Figure 4c, respectively). It remains to be determined with future crop harvests if protease treatment can generally be applied to switch phytoglycogen extracts from a cytotoxic or cytocompatible state into an energy source. 

**Figure 6:**
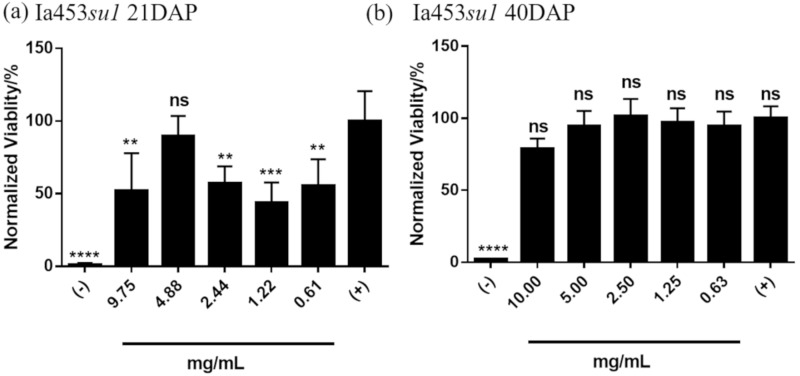
Cytotoxicity of phytoglycogen extracts isolated at different times post-pollination. Phytoglycogen was extracted using ethanol precipitation 21 days after pollination (“Ia435*su1* 21DAP”) or 40 days after pollination (“Ia435*su1* 40DAP”). Cells were treated with 50 *v*/*v* % PBS (positive control) or 50 *v*/*v* % DMSO (negative control). Data are presented as mean ± standard deviation (*n* = 5). * indicates statistically significant differences compared to the positive control group; “ns” indicates no difference relative to the positive control group at t = 24 h. Statistical significance was calculated using a one-way ANOVA followed by Tukey’s post-hoc. ** *p* < 0.01, *** *p* < 0.001 and **** *p* < 0.0001.

Finally, we compared the cytotoxicity of phytoglycogen extracts from Ia453-*su1* from which kernels were processed at different times post-pollination. Unexpectedly, kernels processed at maturity (i.e., 40 days after pollination (“DAP”)) yielded phytoglycogen extracts that were non-toxic to NIH3T3 fibroblasts at all concentrations tested (Figure 6b), whereas extracts from kernels processed at 21 days after pollination were cytotoxic (Figure 6a). This contrasted with non-toxic phytoglycogen extracts from A632*su1*-SW and A632*su1*-Pu, which were extracted 21 days after pollination. It is important to note that the Ia453*su1* kernels were from plants grown in different environments (FL and WI), and the difference observed in cytotoxicity of phytoglycogen extracted at 21 DAP and 40 DAP could be due to environmental effects. Thus, these data suggest that pollination time and/or growth location may be determinants of phytoglycogen extract cytotoxicity. 

### 2.4. Water-Binding Properties of Phytoglycogen Extracts from Different Sources

**Figure 7:**
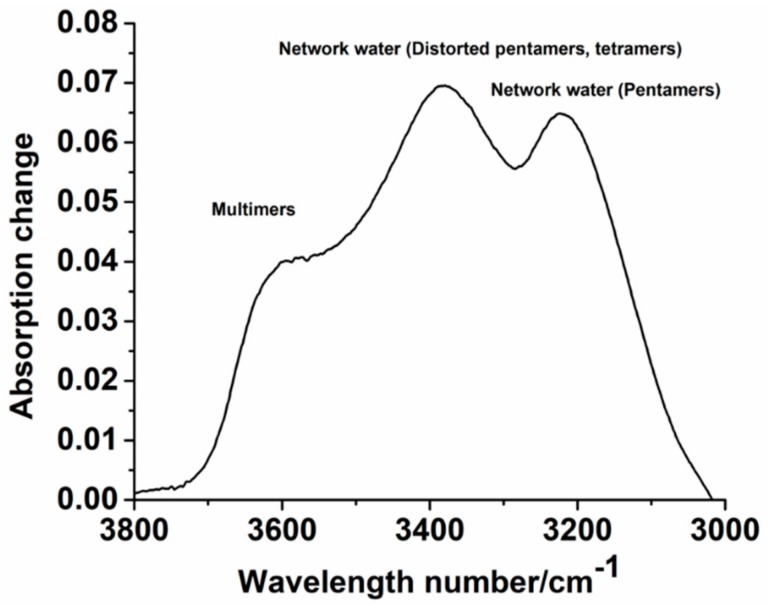
Representative subtracted FTIR spectrum. The peaks located at ~3225, 3400, and 3600 cm^−1^ are used to determine the R_network_/R_multimer_. Spectra for all phytoglycogen extracts can be found in Supplementary Figure S1.

**Figure 8:**
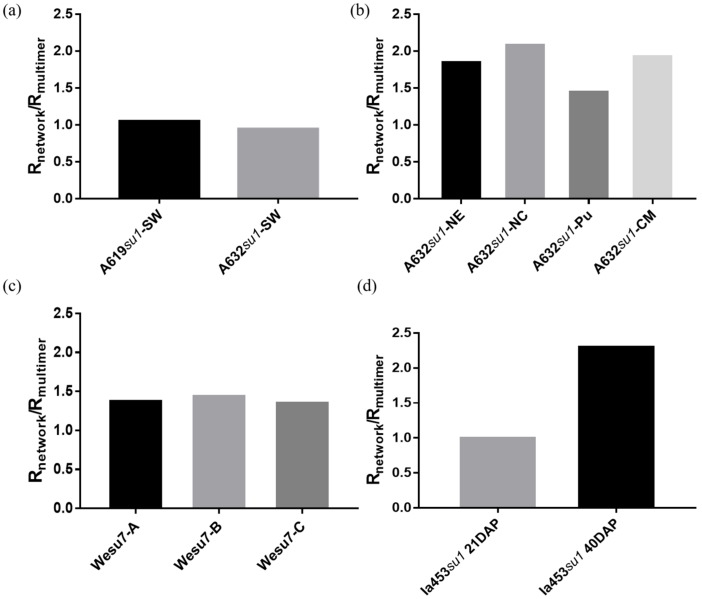
R_network_/R_multimer_ for (**a**) A619*su1*-SW and A632*su1*-SW, (**b**) A632 with different *su1* mutants, (**c**) Wesu7-A, Wesu7-B, and Wesu7-C, and (**d**) Ia453*su1* harvested 21 days after pollination (“Ia435*su1* 21DAP”) or at 40 days after pollination (“Ia435*su1* 40DAP”).

The hydration state of polysaccharides, including phytoglycogen, can vary based on their chemical composition [49]. The organization of water molecules around phytoglycogen could aid in understanding and predicting its interactions with biomolecules or cells in physiological conditions [50]. Here, we postulated that the hydration state of phytoglycogen may correlate with cytotoxicity. To measure the water order around phytoglycogen, we collected FTIR spectra of dry and wet extracts, and compared the R_network_/R_multimer_ ratio, which is determined from the integrated signal intensity at different wavenumbers (Figure 7 and Figure S1). A619*su1*-SW and A632*su1*-SW had similar R_network_/R_multimer_ ratios, which suggested that their observed differences in cytotoxicity are likely due to features of the plant type (Figure 8a). Among A632 *su1* mutants, the R_network_/R_multimer_ ratios varied. We noted that A632 *su1* mutants with R_network_/R_multimer_ greater than 1.5 were cytotoxic to fibroblasts at high concentrations, whereas those with R_network_/R_multimer_ less than 1.5, were non-toxic or stimulated growth (Figure 8b). All extracts from Wesu7 had similar R_network_/R_multimer_ values, regardless of whether they were extracted with ethanol, ethanol and deproteination, or ethanol and protease treatment (Figure 8c). These observations, taken together with DLS measurements, suggested that the extraction method does not affect the physical properties of phytoglycogen itself, but instead that additional processing steps may remove impurities that are cytotoxic to NIH3T3 cells. In contrast, the R_network_/R_multimer_ of the two Ia453*su1* extracts were significantly different from each other, with the cytotoxic extract obtained 21 days after pollination having a smaller ratio than the non-toxic extract obtained ~40 days post-pollination (Figure 8d). These observations suggest that time post-pollination may alter the structure or composition of phytoglycogen in a way that leads to changes in hydration state. Collectively, these observations demonstrate that hydration state is not a reliable predictor of phytoglycogen cytotoxicity in general, although differences in water order between phytoglycogen extracts from A632 or Ia453 variants did correlate with cytotoxicity. 

### 2.5. Contaminating Protein Impurities in Phytoglycogen Extracts from Different Sources

**Figure 9:**
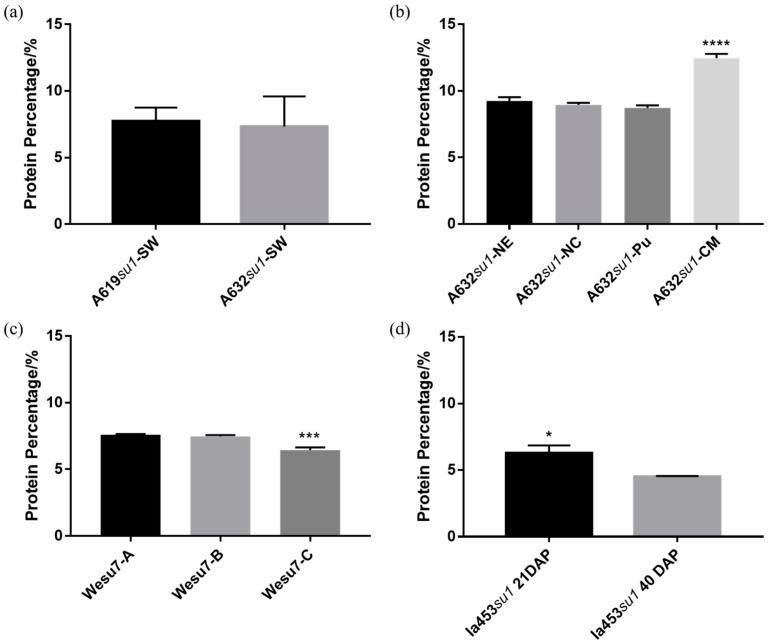
Protein content of phytoglycogen extracts determined using the BCA assay. (**a**) A619*su1*-SW and A632*su1*-SW, (**b**) A632 with different *su1* mutants, (**c**) Wesu7-A, Wesu7-B, and Wesu7-C, and (**d**) Ia453*su1* extracted at 21 days or 40 days post-pollination. Data are presented as mean ± standard deviation (*n* = 3). Statistically significant differences between the means were calculated using an unpaired Student’s t test for (**a**) and (**d**). For (**b**) and (**c**) data were analyzed with one-way ANOVA followed by Tukey’s post-hoc. * *p* < 0.05, *** *p* < 0.001 and **** *p* < 0.0001.

In all phytoglycogen samples, a peak was observed in FTIR spectra within the range of ~1700 cm^−1^ to 1500 cm^−1^, which is associated with the vibration of amide bonds of proteins [51] (Figures S2 and S3). Because protein quantification based on amide FTIR signal can vary due to sample preparation, we subjected each extract to the BCA assay to quantify the amount of protein contaminants (Figure 9). A632*su1*-SW and A619*su1*-SW had a similar percentage of protein impurities (Figure 9a). A632*su1*-CM had a significantly higher protein content than all other A632 *su1* mutants (Figure 9b). Wesu7 extracts treated with protease had lower protein content than extracts obtained with ethanol precipitation alone or those that were deproteinated with acid (Figure 9c). These observations suggested that protease treatment removed protein impurities more effectively than acidic deproteination. Phytoglycogen extracted from Ia453*su1* 40 days post-pollination had a lower protein contaminant concentration than extracts obtained 21 days post-pollination (Figure 9d). In many instances, protein contaminant concentration correlated with cytotoxicity. In particular, A632*su1*-CM was the most toxic extract from this plant and also had the highest protein contaminant concentration. Wesu-7C, which was extracted with ethanol and treated with protease, had the lowest protein contaminant concentration and promoted cell growth when compared to other Wesu-7 extracts. Ia453*su1* extracts obtained 40 days post-pollination had a lower protein contaminant concentration and lower cytotoxicity than extracts obtained after 21 days. Collectively, these observations suggest that protein impurities may contribute to the cytotoxicity profile of phytoglycogen extracts.

## 3. Materials and Methods

### 3.1. Phytoglycogen Extract Sources

In this experiment, we characterized five naturally-occurring *sugary-1* alleles introgressed into one field corn line (A632) plus another 2 lines containing the *su1-*NE mutation (Wesu7 and Ia453) and one line (A619) containing the *su1-*SW allele. The sweet corn kernels utilized in this study were grown in Wisconsin and harvested for another experiment previously described [52]. Kernels were harvested and frozen 21 days after pollination, with exception of sample “Ia453*su1* 40 DAP”, which was grown in Florida in the fall of 2017 and harvested at maturity, 40 days after pollination. 

### 3.2. Phytoglycogen Extraction and Purification

A: Ethanol (EtOH) precipitation method: Phytoglycogen extractions were performed as reported before [32]. Briefly, a volume of water equivalent to six times the weight of each sample of either lyophilized powdered kernels or fresh frozen kernels was added. Frozen kernels were pulverized using a mortar and pestle, while powdered kernels were vortexed for 30 seconds. The suspensions were then passed through a 50 µm filter. Three volumes of 100% EtOH was added to each sample and centrifuged at 8000× *g* for 20 min. The supernatant was decanted and the pellet re-dissolved in 30 mL of 100% EtOH and centrifuged at 8000× *g* for 10 mins. This wash was repeated 2 times. The pellet was then dried under vacuum and maintained in a 60 °C oven for 30 min until powders were completely dry. 

B: Ethanol precipitation with deproteinization: Phytoglycogen extractions were performed as described before [38]. Briefly, 10 g of kernels were ground in 60 mL of cold H_2_O with a mortar and pestle. The homogenates were collected and passed through a 50 µm disposable filter. The solid was further extracted twice using deionized water. The combined liquid was adjusted to pH 4.9 with acetic acid and placed at 4 °C for 2 h to induce protein precipitation. The liquid was then centrifuged at 10,000× *g* at 4 °C and the creamy layer and precipitate were removed. The supernatant was adjusted to pH 7.0 and autoclaved at 121 °C for 60 min. The supernatant was collected and 3 volumes of ethanol were added to precipitate polysaccharides. The precipitate was washed with three cycles of ethanol. After removing the bulk of ethanol by filtration, the solid material was dried in an oven at 37 °C to remove residual ethanol and powdered in a mortar and pestle. 

C: Ethanol precipitation and protease treatment: Phytoglycogen extractions were performed as reported before [46]. Five grams of kernels were ground in 25 mL of 250 mM Tricine buffer, pH 7.5, with protease (2.5 units/mL; bacterial type XIV, Sigma–Aldrich, Castle Hill, NSW, Australia) at 37 °C for 30 min. To this solution, 25 mL cold Tricine buffer was added followed by centrifugation at 4000× *g* for 10 min. The supernatant was collected and 4 volumes of absolute EtOH added. The suspension was then centrifuged for 10 mins at 4000× *g*. The pellet was then re-dissolved in 15 mL DMSO containing 0.05% (*w*/*w*) lithium bromide and incubated at 80 °C in a water bath overnight. The supernatant was collected and 4 volumes of EtOH added, followed by centrifugation. Residual EtOH was removed by filtration and dried in an oven at 37 °C for 30 mins.

The protein content of each phytoglycogen extract was determined using the BCA assay (ThermoFisher Scientific) by adapting procedures reported previously [49]. 

### 3.3. Dynamic Light Scattering

For DLS measurements, all phytoglycogen extracts reported in Table 2 were purified using ethanol precipitation. All phytoglycogen extracts were dissolved in 1 mL of distilled water at a weight to volume ratio of 10 mg/mL. Samples were then sonicated for 30 min in a water bath sonicator. Particulate matter was removed by centrifugation. Samples were then diluted 5 times in 1.25× PBS followed by filtration through a 0.2 µm filter. 1 mL of each sample was then run on a Malvern Zetasizer Ultra (Malvern Instruments Ltd., UK). A refractive index of 1.33457 was used based on dextrin. Each sample was run 3 times and the mean and standard deviation are reported. The polydispersity index is also reported.

### 3.4. Cell Toxicity Using Fibroblasts

For in vitro cytotoxicity experiments, phytoglycogen extracts were dissolved in PBS at 20 mg/mL and vortexed vigorously for 5 min before placing in a sonicating water bath for 30 min. The final solution was sterilized by passing it through a 0.22 µm EMD Millipore™ Millex™ (Millipore Sigma, Burlington, MA, USA) sterile syringe filter. The final phytoglycogen solutions were obtained as milky transparent stocks and were diluted as reported.

The toxicity of different phytoglycogen extracts was evaluated by measuring the metabolic activity of NIH3T3 fibroblasts in vitro using the CellTiter-Blue® reagent (PR-G8080, Promega, Madison, WI, USA), similar to methods reported previously [53,54]. Cells were maintained at 37 °C and 5% CO_2_ in Dulbecco’s Modified Eagle Medium (DMEM) supplemented with 10% (*v*/*v*) fetal bovine serum (Biochrom, Berlin, Germany), 1% penicillin-streptomycin, and 1% (*v*/*v*) l-glutamine, referred to as “complete fibroblast media”. Before cell seeding, cells were harvested using trypsin-EDTA (0.25% trypsin) and counted with a hemocytometer (Fisher Scientific, Hampton, NH, USA). Then cells were aliquoted into a clear tissue culture-treated 96-well microplate (20,000 cells per well) in 50 µL of complete fibroblast media. For experimental groups, cells were treated with 50 µL of sterile filtered solutions of phytoglycogen extracts in PBS at various dilutions prepared from each stock. For negative and positive controls, cells were treated with 50 µL PBS or DMSO, respectively. Cells were incubated at 37 °C for 24 h, at which point the culture media was aspirated and replaced with 100 µL fresh complete fibroblast media. 20 μL of CellTiter-Blue® reagent (PR-G8080, Promega) was then added to each well. Fluorescence emission of each well was measured using a SpectraMax M3 plate reader (excitation = 560 nm, emission = 590 nm) at 1, 2, 3, and 4 h.

### 3.5. FTIR Spectroscopy

The interaction between different phytoglycogen extracts and water was investigated using FTIR spectroscopy by adapting methods reported previously [49]. In brief, 1 mg of phytoglycogen extract (dry powder) was deposited onto a ZnSe-diamond ATR crystal. Spectra were collected on a Bruker Vertex 70 FTIR spectrometer equipped with an MCT-A detector using an incident angle of 45°. Spectra were acquired at a resolution of 4 cm^−1^. Spectra were collected both before and after adding 0.1 µL ultra-pure water to dry phytoglycogen extract samples. Water organization around phytoglycogen extracts was calculated by quantifying spectral changes, according to methods reported previously [49]. In short, each water band was calculated by integrating a 40 cm^−1^ window from the component centers (e.g. 3225, 3400, and 3600 cm^−1^) with Fourier self-deconvolution. The integrated area is proportional to the number of absorbing water molecules in the spectral range, where A_3600_ corresponds to multimer, A_3400_ corresponds to distorted pentamers or tetramers, and A_3225_ corresponds to pentamers [55]. Using these absorbance values, the spectral parameters R_network_ (R_network_ = A_3225_/A_3400_) and R_multimer_ (R_multimer_ = A_3600_/A_3225_) were calculated for each phytoglycogen extract. These parameters provide information about the relative populations of the different water types and are known to be sensitive to the structural arrangement of hydrogen bonds [55,56,57,58,59,60,61].

### 3.6. Statistical Analysis 

All cell experiments were conducted with five technical replicates. Data are reported as mean and standard deviation. Statistically significance differences between data points were determined using a one-way ANOVA followed by Tukey’s post-hoc or Student’s t-tests in GraphPad Prism software (GraphPad Software, San Diego, CA, USA).

## 4. Conclusions

This study reports the characterization of the cytocompatibility of phytoglycogen extracts from different maize sources. Phytoglycogen extracted from A632*su1*-SW was not cytotoxic, whereas phytoglycogen from A619*su1*-SW was. Among extracts from A632 varieties with different *su1* mutants, A632*su1*-NE, -NC, and -CM were cytotoxic, whereas A632*su1*-Pu promoted cell growth. Changing the extraction methods improved the cytocompatibility of phytoglycogen extracts from Wesu7, whereas changing the harvest time improved the cytocompatibility of extracts from Ia435*su1*. Hydrodynamic diameter and solubility did not correlate with cytotoxicity in any case. Within A632, Wesu7, and Ia435 plant varieties, the concentration of protein contaminants correlated with cytotoxicity. Among A632 and Ia435 varieties, water-binding properties also correlated with cytotoxicity. Although the mechanism by which phytoglycogen extracts induce cell death remains unknown, collectively these data demonstrate that phytoglycogen extracts are not uniformly cytocompatible. Rather, these data demonstrate that the maize variant, plant genotype, protein contaminants, and water-binding properties are determinants of the cytocompatibility of phytoglycogen extracts. Thus, future efforts that employ maize phytoglycogen extracts for medical or biotechnology applications should consider the source and processing methods when evaluating safety and toxicity.

## Figures and Tables

**Table 1:** Solubility of phytoglycogen extracts in 1× phosphate-buffered saline.

Variants	Concentration (mg/mL) ^a^	Extraction Method
A619*su1*-SW	20.17 ± 1.37	Ethanol precipitation
A632*su1*-SW	15.41 ± 1.46	Ethanol precipitation
A632*su1*-NC	18.41 ± 2.51	Ethanol precipitation
A632*su1*-Pu	17.18 ± 3.28	Ethanol precipitation
A632*su1*-NE	19.56 ± 2.8	Ethanol precipitation
A632*su1*-CM	18.01 ± 3.18	Ethanol precipitation
Wesu7-A (*su1*-NE)	19.75 ± 1.54	Ethanol precipitation
Wesu7-B (*su1*-NE)	20.41 ± 0.54	Ethanol precipitation + deproteination
Wesu7-C (*su1*-NE)	20.26 ± 1.43	Ethanol precipitation + protease
Ia453*su1* 21DAP	19.51 ± 2.76	Ethanol precipitation
Ia453*su1* 40DAP	20.00 ± 1.34	Ethanol precipitation

^a^ Extract concentrations were measured in 1× PBS. Data are presented as mean ± standard deviation of three independent replicates. DAP = days after pollination. Means were analyzed for statistically significant differences using one-way ANOVA with Tukey’s post-hoc.

**Table 2:** Hydrodynamic diameter and polydispersity of phytoglycogen extracts isolated using ethanol precipitation.

		Diameter/nm	PDI
A	A619*su1*-SW	77.5 ± 0.63	0.089
B	A632*su1*-SW	74.8 ± 0.096	0.103
C	A632*su1*-NE	75.2 ± 0.371	0.124
D	A632*su1*-NC	66.5 ± 0.643	0.075
E	A632*su1*-Pu	70.8 ± 0.842	0.096
F	A632*su1*-CM	76.8 ± 0.36	0.111
G	Wesu7-A (*su1*-NE)	68.1 ± 1.09	0.135
H	Ia453*su1* 21DAP	77 ± 0.53	0.082
I	Ia453*su1* 40DAP	74.7 ± 1.170	0.123

Data collected in 1× PBS. All data reported are based on intensity-weighted mean hydrodynamic diameters. Data are reported as mean ± standard deviation of three independent replicates. DAP = days after pollination.

## Supplementary Materials

The following are available online. Figure S1: FTIR spectra of water binding to phytoglycogen, Figure S2: FTIR spectra of dry phytoglycogen extracts, Figure S3: FTIR spectra of phytoglycogen protein contaminants, Table S1: hydrodynamic diameters of Wesu7 extracts (*su1*-NE) with different processing steps.
