# Supplementary material for: Characterizing the Physical Properties and Cell Compatibility of Phytoglycogen Extracted from Different Sweet Corn Varieties
Source: Molecules. 2020 Feb 1;25(3):637. doi: 10.3390/molecules25030637 (PMC7037141; doi:10.3390/molecules25030637)
Supplement: Supplementary file 1 [file molecules-25-00637-s001.pdf]

Supplementary information

Characterizing the physical properties and cell compatibility of phytoglycogen extracted from different sweet corn varieties

Renjie Liu[1], Susan K. Boehlein[2], William F. Tracy[3], Marcio F. R. Resende Jr.[2, *], and Gregory A. Hudalla[1, *]

[1] J. Crayton Pruitt Family Department of Biomedical Engineering, Wertheim College of Engineering, University of Florida, Gainesville, FL 32611, USA

[2] Horticultural Sciences Department, University of Florida, Gainesville, FL, 32611, USA

[3] Department of Agronomy, University of Wisconsin-Madison, Madison, WI, 53706

* indicates corresponding authors

Author contact:

Gregory A. Hudalla, PhD
Biomedical Sciences J293, PO BOX 116131
1275 Center Drive
Gainesville, FL 32611
ghudalla@bme.ufl.edu
(352) 273-9326

Marcio F. R. Resende Jr.
Fifield Hall 2135, PO BOX 110690
2550 Hull Road
Gainesville, FL 32611
mresende@ufl.edu
(352) 273-4772

Table S1. Summary of average hydrodynamic diameter of Wesu7 extracts (*su1*-NE) with different processing steps.

	Diameter	PDI	Extraction method
Wesu7-B (*su1*-NE)	62.8 $\pm$0.986	0.249	Ethanol precipitation + deproteination
Wesu7-C (*su1*-NE)	61.8	0.217	Ethanol precipitation + protease treatment

Data collected in 1X PBS; all data reported are based on intensity-weighted mean hydrodynamic diameter.

Figure S1. Subtracted FTIR spectra of phytoglycogen extracts over the range of 3800 cm^{-1} to 3000 cm^{-1} (a) A619*su1*-SW and A632*su1*-SW, (b) A632 with different *su1* mutants, (c) Wesu7-A, Wesu7-B and Wesu7-C with different extraction-purification methods, and (d) Ia453*su1* with different harvest times.

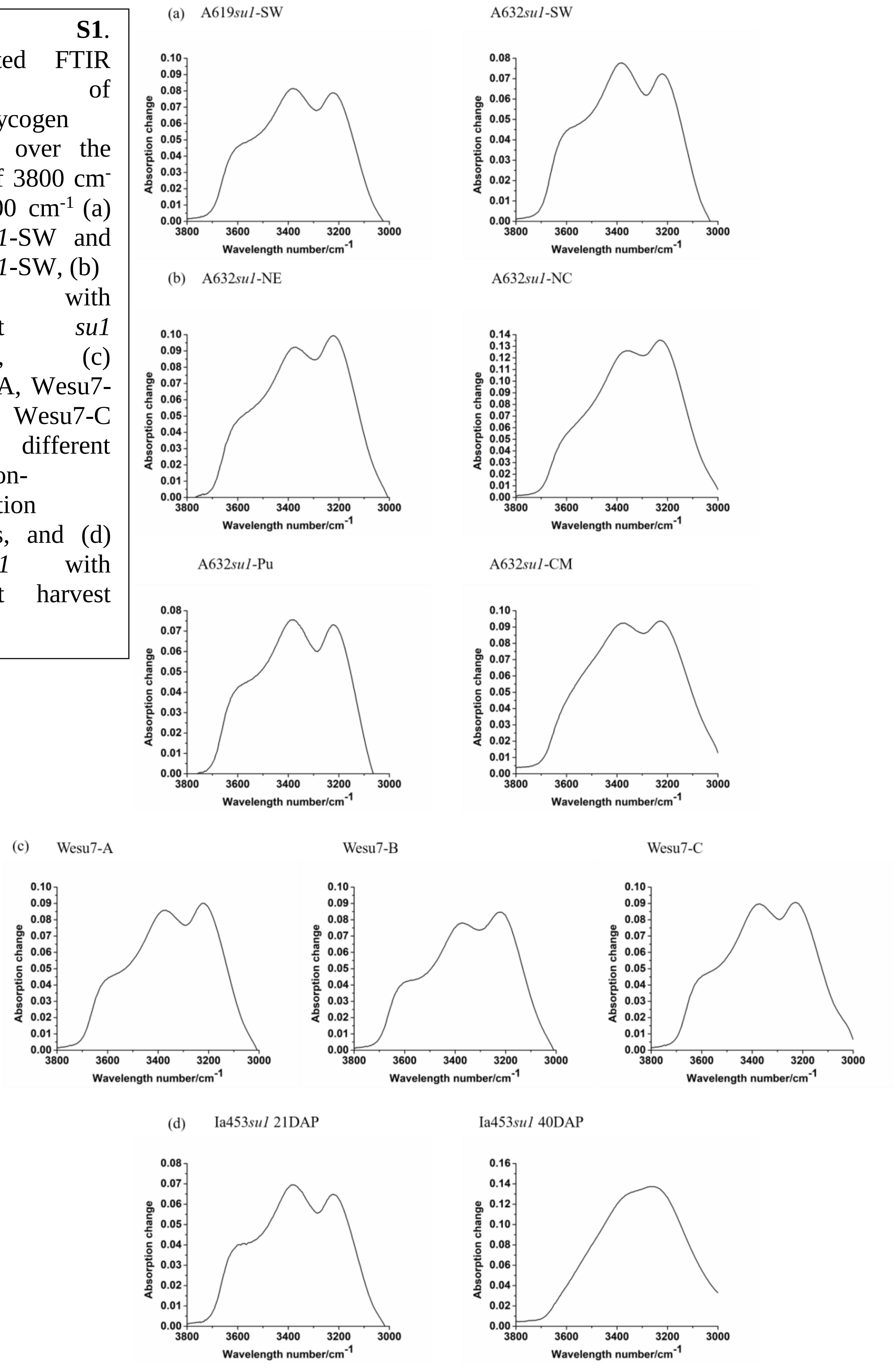

Figure S2. FTIR spectra of phytoglycogen extracts (a) A619*su1*-SW and A632*su1*-SW, (b) A632 with different *su1* mutants, (c) Wesu7-A, Wesu7-B and Wesu7-C with different extraction-purification methods, and (d) Ia453*su1* with different harvested times.

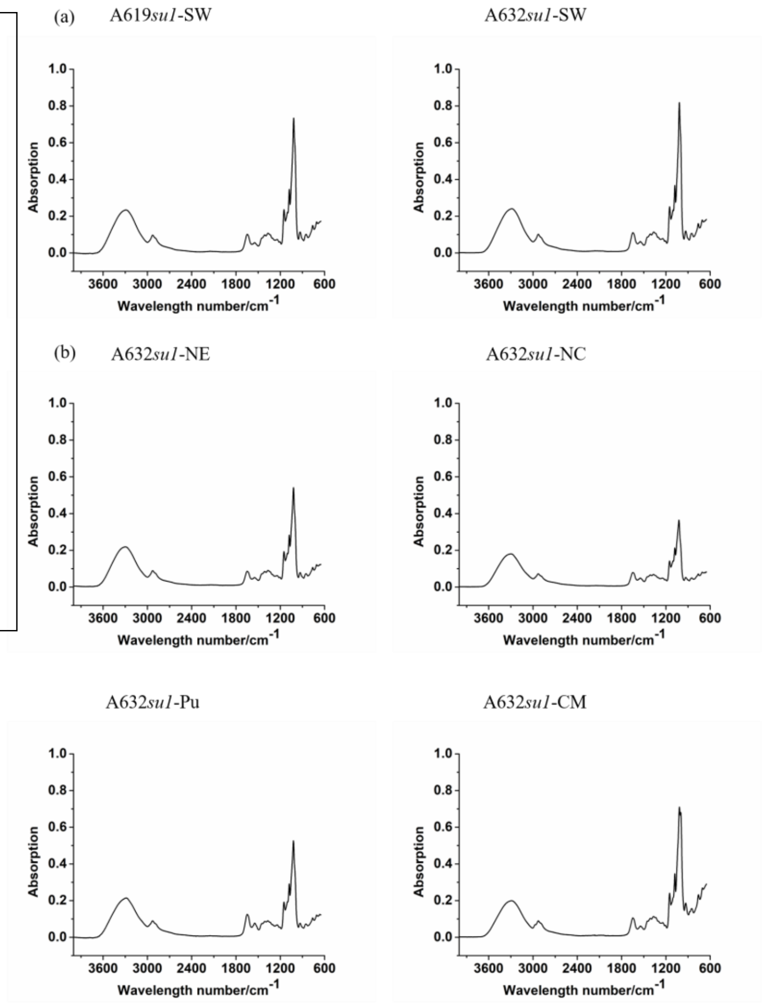

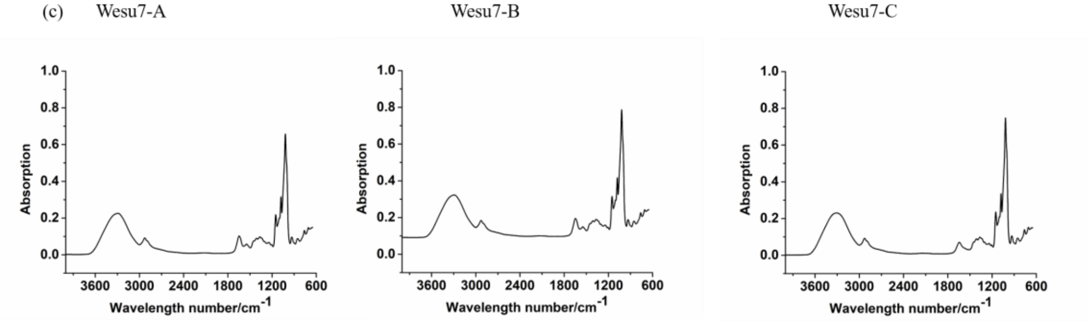

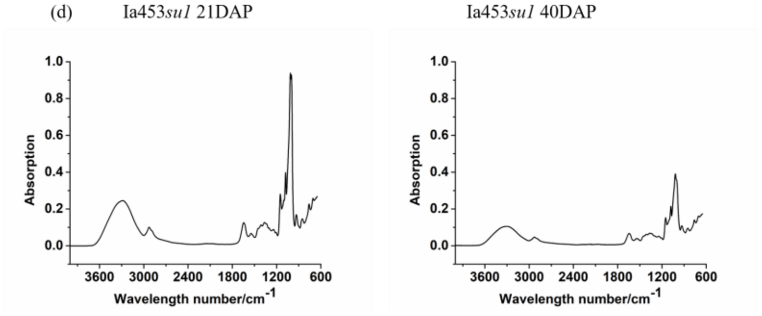

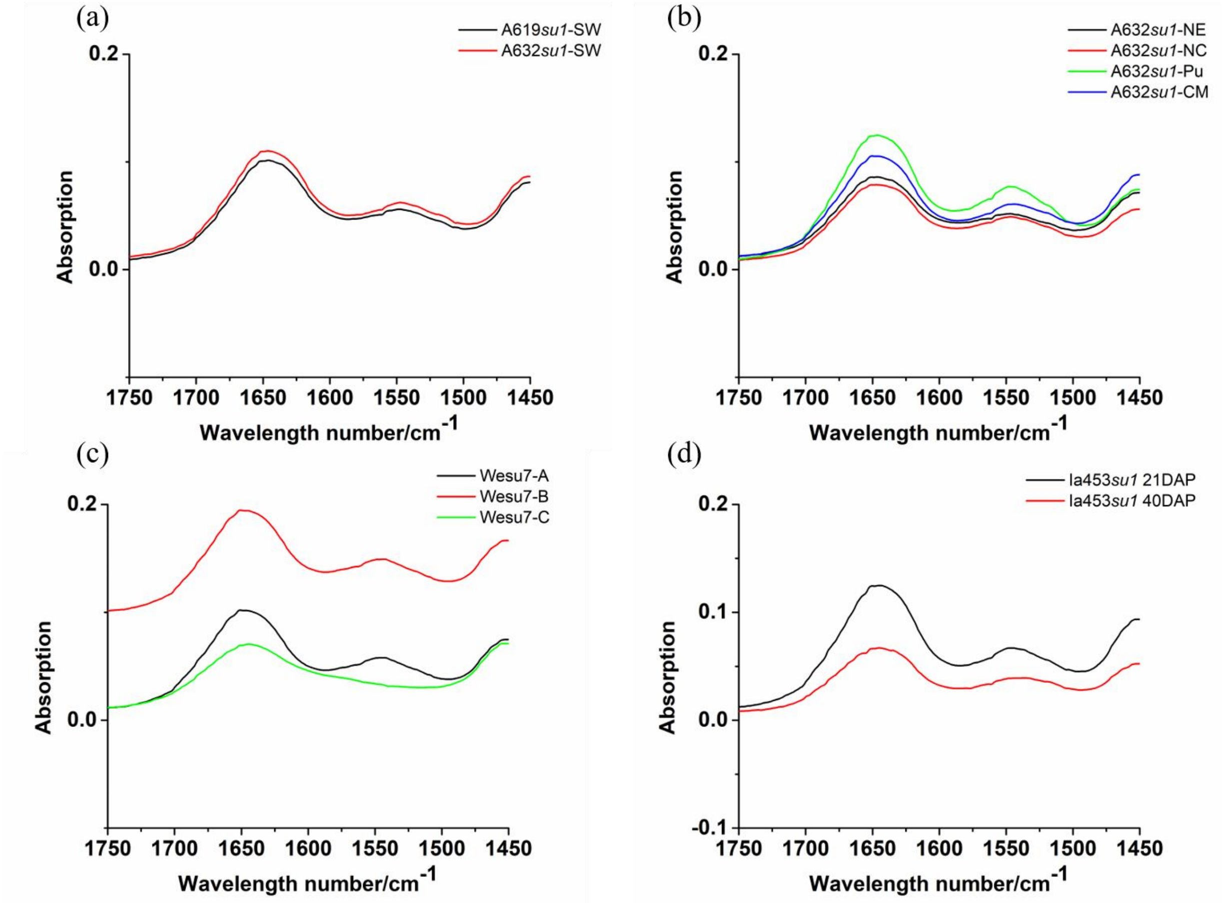

Figure S3. FTIR spectra of dry phytoglycogen extracts over the range of 1750 cm^{-1} to 1450 cm^{-1} (amide I) (a) A619*su1*-SW and A632*su1*-SW, (b) A632 with different *su1* mutants, (c) Wesu7-A, Wesu7-B and Wesu7-C with different extraction-purification methods, and (d) Ia453*su1* with different harvested times.
